# Subtle outer retinal and choroidal alterations in patients at high risk of progression to age-related macular degeneration

**DOI:** 10.3389/fmed.2026.1807534

**Published:** 2026-04-28

**Authors:** Lulu Bao, Haihang Ying, Xiaomin Wang, Miaoqin Wu, Hui Liu

**Affiliations:** 1 Department of Ophthalmology, Rehabilitation Medicine Center, Zhejiang Provincial People’s Hospital, Affiliated People’s Hospital of Hangzhou Medical College, Hangzhou, Zhejiang, China; 2 Yongkang Hospital, Jinhua, Zhejiang, China

**Keywords:** AMD, choroidal thickness, OCT, OCTA, outer retina

## Abstract

**Aim:**

To explore the changes in the microstructure and blood supply of the outer retina in eyes at high risk of progression to age-related macular degeneration (AMD).

**Methods:**

Forty-seven patients with unilateral neovascular AMD (nAMD) were enrolled. Twenty-two of the contralateral eyes were considered at high risk of progression to nAMD (Group 1), while the remaining 25 eyes had dry AMD (Group 2) Fifty healthy subjects (50 eyes) were enrolled as control. Swept-source optical coherence tomography (SS-OCT) equipped with angiovue (OCTA) was used to obtain three-dimensional retinal thickness maps and microvascular images of the superficial and deep retinal capillary plexuses (SCP and DCP) around the macula and choroid vessel index (CVI). Quantitative analysis was automatically calculated by an inbuilt algorithm in the SS-OCTA (VG200; SVision Imaging, Ltd., Luoyang, China). One-way analysis of variance (ANOVA) was used to compare the differences among the groups, and *post hoc* procedures were used to compare differences between the two groups. Associations between OCTA-derived parameters and retinal/choroidal thickness were evaluated using Pearson correlation coefficients.

**Results:**

Compared to the controls, the densities of the SCP and DCP were significantly decreased in Group 2 in some regions (*p* < 0.05). However, the CVI of patients in superior (S) and temporal (T) regions was significantly decreased compared to the controls (*P*: 0.009, 0.020). The outer retinal thickness of Group 2 in the central (C), S, and temporal (T) regions were significantly decreased compared to the controls and Group 1 (*P*: 0.004–0.048). Meanwhile, compared to the controls, the thickness of total retina and choroid of Group 2 were significantly decreased in most regions (*P*: 0.001–0.034). The outer retinal thickness was significantly correlated to choroidal thickness in AMD patients (*r* = 0.346, *p* = 0.034).

**Conclusion:**

Significant thinning of outer retina and choroid were observed in patients with dry AMD. In eyes at high risk of progression to AMD, choroidal thickness and CVI tend to be decreased. SS-OCTA might be useful in evaluating microstructural and blood supply disorders of the outer retinal layers in healthy eyes of unilateral nAMD patients, which might be helpful to identify the earliest progression of AMD.

## Introduction

Age–related macular degeneration (AMD) is the third leading cause of blindness worldwide and prevalence increases with age (1, 2). Patients with AMD in China are concentrated in the 85–89 age range, and the number of patients has increased from 1.201 billion in 1990 to 2.665 billion in 2015, while the number of patients with early AMD increased to 2.091 billion (3). Causes of AMD are complex and include metabolic, environmental, functional, and genetic factors. The death of retinal pigment epithelial (RPE) cells, choroidal endothelial cells (CECs), and photoreceptor cells is common in advanced AMD. Loss of CECs is one of the earliest detectable events in AMD and may be the initial point for progression to the advanced stages of AMD, as choriocapillaris offer metabolic support to the outer retina (4, 5). Lipofuscin and cell debris of photoreceptor cells can normally be expelled by RPE and then cleared by capillaries in pathological conditions. Previous studies found that the onset site of AMD is Bruch’s membrane (BM) (6). When RPE cell function is abnormal, permeability changes in BM lead to the gradual accumulate of lipofuscin and cell debris between RPE and BM to form drusen (7), which is related to the thickening and calcification of the BM collagen layer. It has also been observed that atrophy of the choroidal layer in AMD patients further reduces the clearance of extracellular substances, thus leading to the formation of drusen (8). Initially, abnormal blood vessels which coexist with subretinal fluid accumulation may appear before degeneration and the formation of a disk scar (8, 9). As a result, irreversible and permanent loss of vision acuity eventually occurs, caused by neovascular AMD (10).

At present, the study of early AMD mainly includes the morphological changes of the outer retina in the macular area, drusen, and choroid (4). The main methods include fundus photograph (11), optical coherence tomography (OCT), fundus spontaneous fluorescence imaging, and indocyanine green angiography (ICGA). OCT equipped angiography (OCTA) is a non-invasive instrument that can quantify the microvascular of retina and choroid in patients with AMD (12–14).

Early AMD is usually asymptomatic, although RPE mottling and extracellular lipofuscin deposits between RPE cells and Bruch’s membrane can be clinically detected in the posterior pole (6). Some studies found significant differences in retinal layers between patients with early AMD and healthy controls (15), while others reported conflicting results (16). However, there are fewer studies on the changes of retinal vascular and structure in early AMD, which is a topic that needs further exploration.

The disease stage of the contralateral eye also has a significant influence on the occurrence and development of AMD. AMD usually develops in both eyes, and the more severe the stage is in the contralateral eye, the faster the disease progresses in the study eye, as described in a 20-year follow-up study (Age-Related Eye Disease Study Severity (AREDS) scale) (17, 18). As reported in large sample clinical studies, 72.4% of patients with unilateral advanced AMD still have good vision in the opposite eye, and the probability of severe vision loss in the opposite eye is 2–30% per year (17). Nichole Joachim et al. found in a 5-year follow-up that a greater proportion of unilateral AMD progressed to bilateral AMD, which was more obvious in unilateral advanced AMD. The incidence of AMD within 5 years in healthy eyes was 2.4–3.5 times higher for unilateral AMD than those without AMD (19). Therefore, in this study, the contralateral healthy eye of patients with unilateral nAMD (AREDS 5-step classification) (18) was considered at high risk of progression to AMD (Group 1). Changes in fundus structure, microvascular, and visual function in Group 1 were observed to seek possible biological markers for early identification of the occurrence of AMD. A previous study showed that subthreshold micropulse laser can alleviate visual loss and possibility of progression to advanced AMD in eyes with intermediate AMD (20). Timely intervention and treatment can delay the course of AMD and avoid irreversible vision loss (21).

## Methods

### Patients

Patients diagnosed with nAMD were retrospectively identified by an ophthalmologist using the international age-related maculopathy (ARM) epidemiological study group grading system (22). The untreated contralateral eye of each patient was then evaluated in detail. Patients with AMD were graded by an ophthalmologist according to the description (0) a, No signs; b, Few hard drusen (<63 μm); (1) a, Soft distinct (>63 μm); b, Pigment irregularities only (no soft drusen); (2) a, Soft indistinct drusen (>125 μm); b, Soft distinct drusen (>63 μm) and pigment irregularities; (3) Soft indistinct drusen (>125 μm) and pigment irregularities; and (4) a, geographic atrophy (GA); b, choroidal neovascularization (CNV); c, GA and CNV (17). Patients were retrospectively selected from the Zhejiang Provincial People’s Hospital, China from May 2022 to May 2023. Inclusion criteria were (1) stable foveal fixation and (2) spherical equivalent (SE) refractive error between −5.00 and +5.00 diopters.

Exclusion criteria were (1) any established retinal or choroidal disease; (2) presence of any drusen-like deposits, atrophy, or retinal detachment on indirect ophthalmology or swept source optical coherence tomography (SS-OCT) examination; (3) ocular trauma or surgery; (4) systemic disorders with ocular involvement such as diabetes, hypertension, and obesity; and (5) a history of smoking, alcohol consumption, or recreational drug use.

The study was performed in accordance with the tenets of the Declaration of Helsinki, and the Institutional Review Board/Ethics Committee of Zhejiang Provincial People’s Hospital approved the study protocol. Age- and sex-matched control subjects were recruited from workers at the Zhejiang Provincial People’s Hospital.

### Evaluations and measurements

#### Clinical examinations

All subjects underwent comprehensive clinical examinations, including spherical equivalent refractive and best corrected visual acuity (BCVA), intraocular pressure (IOP) measurement (Kowa applanation tonometer HA-2; Kowa Company Ltd. Tokyo, Japan), slit-lamp biomicroscopy, and ophthalmoscopy. In addition, fundus photography was performed with a 200-degree ultrawide field color fundus photograph (Optos PLC, Dunfermline, Scotland, UK).

#### Image acquisition protocol and analysis

All subjects were imaged using a commercial SS-OCTA device (VG200; SVision Imaging, Ltd., Luoyang, China). The SS-OCTA has a central wavelength of 1,050 nm. The 6 × 6 mm scan patterns centering on the fovea were used in this study. Images with a high signal strength index (>7) were evaluated further.

**Figure 1:**
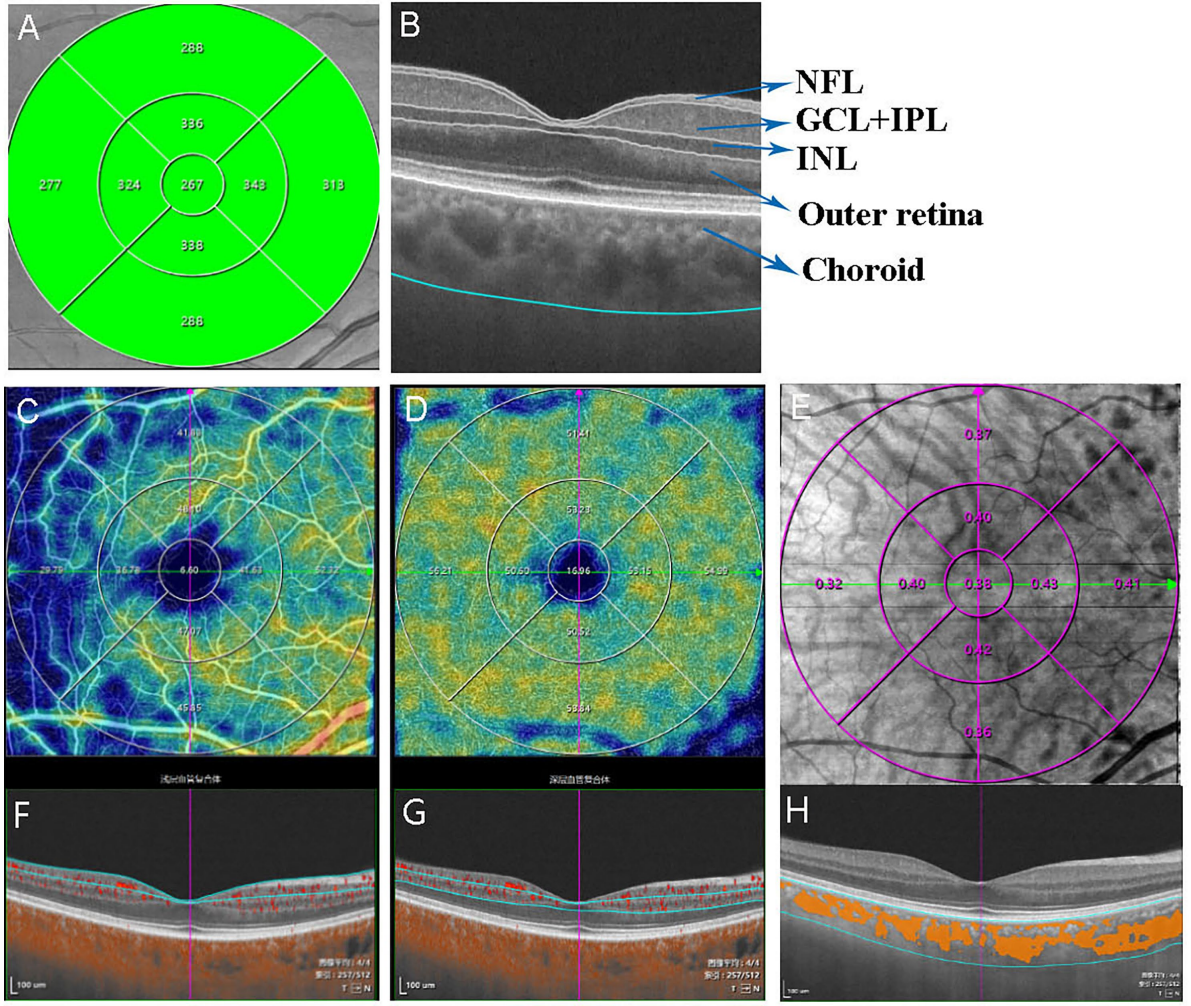
Representative OCTA images used in analysis. **(A)** The total retinal thickness map, **(B)** Five layers of the Intra-retinal and choroid structures in horizontal scan OCT images: NFL, GCL+IPL, INL, Outer retina, and choroid, **(C)** superficial capillary plexus slab, **(D)** deep capillary plexus slab, **(E)** choroid vessel index (orange overlay), **(F)** The SCP was defined as the microvasculature between the base of the retinal nerve fiber layer (RNFL) to the junction between the inner plexiform layer (IPL) and inner nuclear layer (INL), **(G)** DCP was defined as the microvasculature between the INL/OPL junction to 25 μm below it, **(H)** The choroid vessel index (CVI) is defined as the ratio of the volume of Haller’s and Sattler’s layers to the volume of the choroid. The updated image has been uploaded separately. Cross-sectional B-scans below each en face image indicate segmentation accuracy. All images were acquired using the SS-OCTA device (VG200; SVision Imaging, Ltd., Luoyang, China).

The inbuilt algorithm in the SS-OCTA was used to segment and measure the thickness of the (1) NFL; (2) GCL + IPL; (3) INL; (4) outer retina (include outer plexiform layer (OPL), Henle fiber layer and outer nuclear layer (HFL + ONL), the myoid and ellipsoid zone (MEZ), photoreceptor outer segments (OS), and interdigitation zone + retinal pigment epithelium (IZ + RPE)); and (5) choroid (Figure 1B). Manual corrections within the SS-OCT were used to adjust segmentation errors. For analysis, the macular thickness map was divided into a 1-mm diameter circle centered over the fovea and a total annular zone (TAZ) surrounded by a concentric ring 0.5–1.5 mm from the fovea. The areas of the central circle and the TAZ were then divided into five regions: the central (C) and the superior, temporal, inferior, and nasal regions (S, T, I, and N, respectively) (Figure 1A).

Parameters of OCTA from the inner circle were automatically calculated in the parafoveal regions, namely S, T, I, and N, of the 3-mm diameter circular zone after excluding the foveal avascular zone (FAZ), while the outer circle was excluded because of an incomplete edge in the 6 × 6 mm scanning area (Figures 1C,D). The retinal capillary density was calculated as the percentage of pixels with microvascular by an automated algorithm to extract the images of the retinal capillary network in the OCTA images. The en face-OCT image was acquired by projecting a 3D angiography volume data onto a 2D imaging plane, which can provide planar images of the retina and choroid at different levels. A custom automated algorithm was performed on the *en face* OCT-A projection images to quantify the superficial capillary plexus (SCP) and deep capillary plexus (DCP) from a 6 × 6 mm scan pattern. The SCP was defined as the microvasculature between the base of the retinal nerve fiber layer (RNFL) to the junction between the inner plexiform layer (IPL) and inner nuclear layer (INL) (Figure 1F). DCP was defined as the microvasculature between the INL/OPL junction to 25 μm below it (Figure 1G). The OCTA parameter was described in a previous study (12). The choroid vessel index (CVI) is defined as the ratio of the volume of Haller’s and Sattler’s layers to the volume of the choroid (Figure 1H), which was automatically measured by an artificial intelligence-based algorithm that identifies Haller’s and Sattler’s layers on B-scans and reconstructs 3D graphic maps of the medium-diameter and large-diameter choroidal vessel layers (23) (Figure 1E).

### Statistical analysis

All data were calculated as means ± standard deviations and analyzed with SPSS software (version 22.0; SPSS, Inc., Chicago, IL, USA). The spherical equivalent (SE) of the refraction error was calculated as the spherical dioptric power plus one-half of the cylindrical dioptric power. Differences of gender among the groups were determined by the *x*^2^ test. One-way analysis of variance (ANOVA) was used to compare differences among the groups, followed by *post hoc* procedures for pairwise comparisons. *p* values of < 0.05 were considered statistically significant. Associations between OCTA-derived parameters and retinal/choroidal thickness were evaluated using Pearson correlation coefficients.

## Results

**Figure 2:**
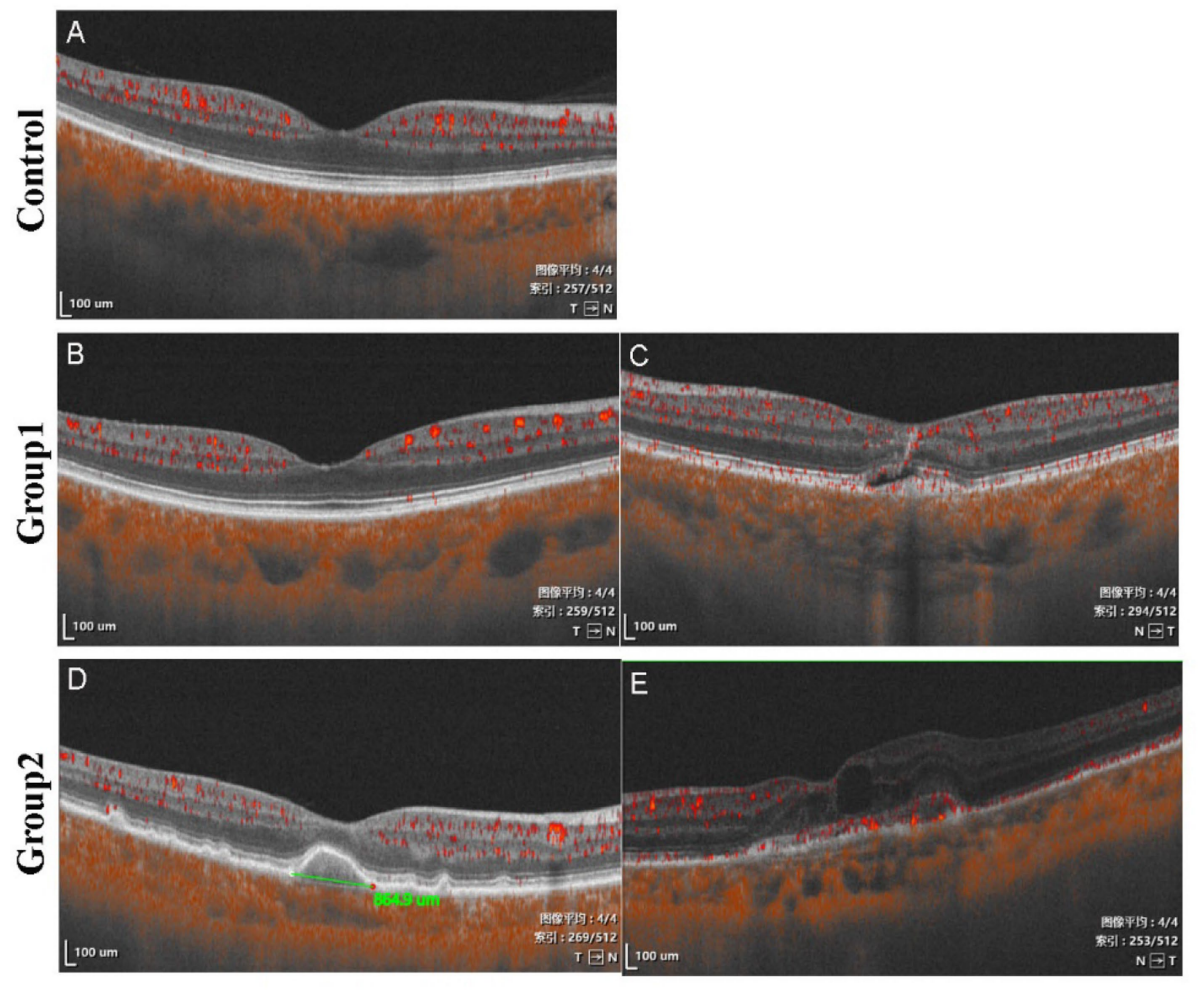
Representative flow signal diagram in an OCT B-scan of a control eye, a Group 1 eye, and a Group 2 eye. The maximum diameter of drusen in dry AMD patient was 864.9 μm (As shown in the green measurement line in the picture).

**Figure 3:**
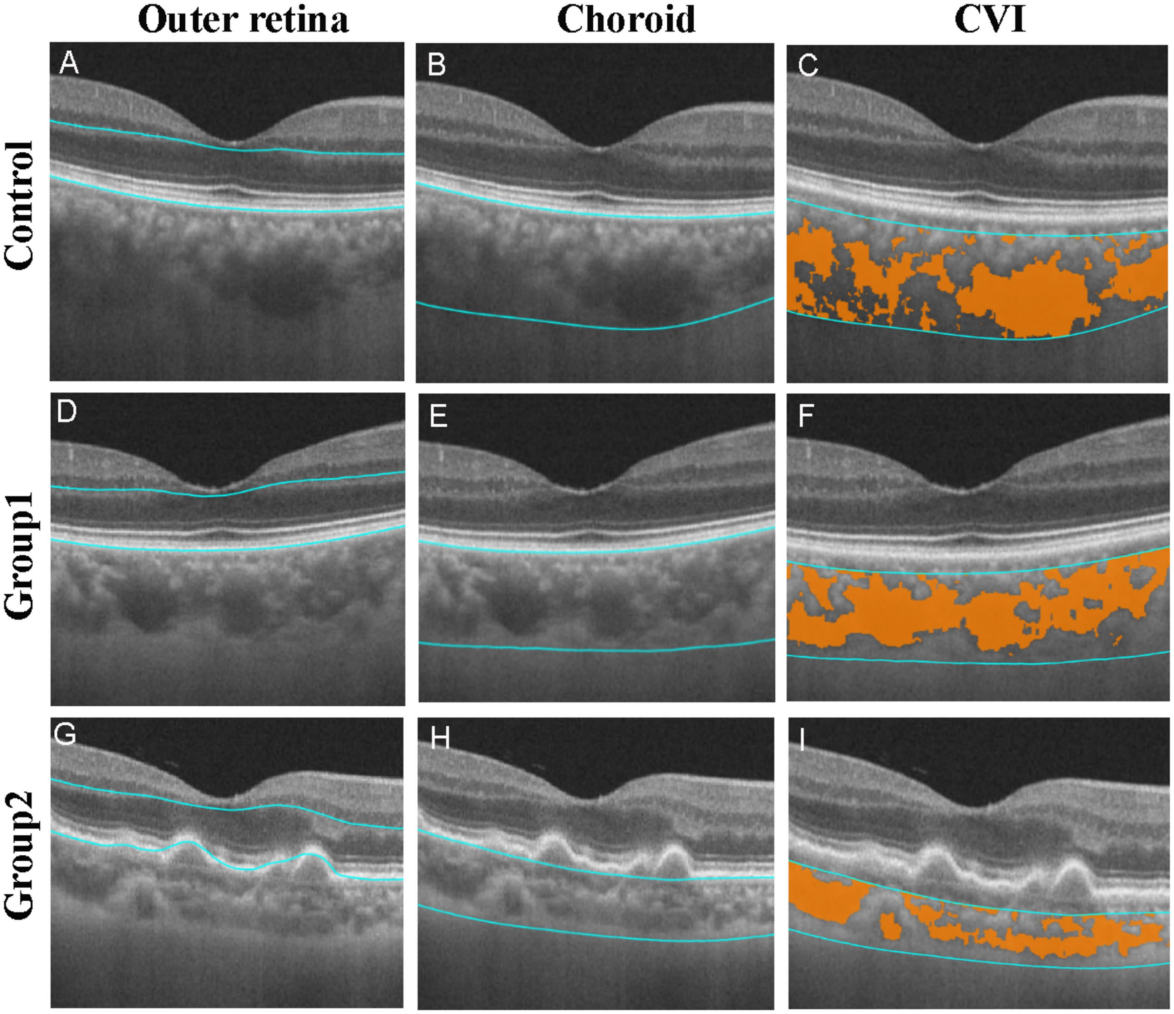
Representative OCT B-scan images of a retina and choroid of a control eye, a Group 1 eye, and a Group 2 eye. The outer retina and choroid of the dry AMD patient was obviously thinner than control. The blue boundary line in Figures **A**, **D**, and **G** represents the outer retina area. The blue boundary line in Figures **B**, **E**, and **H** represents the choroidal area. The blue boundary line is shown in Figures **C**, **F**, and **I**, representing the choroidal vascular area. The orange area represents the volume of Haller’s and Sattler’s layers.

In total, 47 patients with nAMD in a unilateral eye were included in our study (Figures 2C,E). According to the stage of the contralateral eye, there were 22 healthy eyes considered at high risk of progression to AMD, which were classified as Group 1 (Figure 2B), and 25 eyes of dry AMD, which were classified as Group 2 (Figure 2D). In addition, 50 healthy subjects (50 eyes) were enrolled as a control group (Figure 2A). There were no significant differences in the age, gender, SE, or IOP among the three groups (*p* = 0.153–0.954, Table 1), except BCVA (*p* = 0.003). There was no significant difference in baseline BCVA between the control group patients and the Group 1 patients (*p* = 0.238). However, the baseline BCVA of Group 2 was significantly lower than that of the control group (*p* = 0.001). Raw OCTA images in the Control, Group 1 and Group 2 eyes were presented in Figure 3. In eyes with dry AMD, the outer retina and choroid was evidently thinner.

**Table 1:** Basic characteristics of the subjects.

Variables	Control	Group 1	Group 2	*p* value
*N* (Eyes)	50 (50)	22 (22)	25 (25)	–
Gender (F:M)	23:27	10:12	12:13	0.919
Age, years	63.3 ± 8.7	65.8 ± 10.6	66.6 ± 10.8	0.153
SE, diopters	−0.31 ± 2.3	−0.37 ± 1.8	−0.16 ± 1.6	0.954
BCVA, LogMAR	0.02 ± 0.07	0.05 ± 0.12	0.12 ± 0.16	**0.003**
IOP, mmHg	14.9 ± 3.3	15.3 ± 2.4	15.2 ± 2.7	0.841

F, female; M, male; SE, spherical equivalent; BCVA, best corrected visual acuity; IOP, intraocular pressure. Bold *p*-value represents < 0.05.

Compared to controls, SCP and DCP densities were significantly reduced in Group 2 in specific regions (SCP: S and T regions; DCP: S region). The CVI of patients in S and T regions was significantly decreased compared to the controls (*p* < 0.05, Table 2).

**Table 2:** The retinal capillary density (%) and choroid vessel index (CVI) among the three groups.

Layers	Regions	Control	Group 1	Group 2	P^*^	*P*^+^	*P*^#^	*p*
DCP	S	52.6 ± 5.1	52.7 ± 5.1	49.2 ± 8.4	0.902	**0.029**	0.054	0.175
	T	52.4 ± 4.1	52.2 ± 5.2	48.5 ± 10.0	0.748	0.050	0.057	0.209
	I	52.4 ± 4.5	50.9 ± 6.8	52.1 ± 5.5	0.611	0.122	0.395	0.582
	N	51.9 ± 5.2	52.3 ± 5.0	49.0 ± 11.3	0.860	0.124	0.096	0.227
SCP	S	44.7 ± 5.4	44.9 ± 10.4	40.4 ± 8.9	0.886	**0.016**	0.059	0.114
	T	36.2 ± 4.7	36.8 ± 8.4	33.0 ± 8.0	0.750	**0.033**	0.132	0.180
	I	45.1 ± 6.3	44.0 ± 11.9	41.8 ± 9.0	0.409	0.260	0.826	0.288
	N	39.3 ± 5.6	39.7 ± 10.8	35.9 ± 9.7	0.793	0.058	0.175	0.288
CVI	C	0.41 ± 0.06	0.40 ± 0.07	0.39 ± 0.05	0.496	0.294	0.832	0.521
	S	0.41 ± 0.06	0.40 ± 0.09	0.36 ± 0.03	0.809	**0.030**	0.140	**0.009**
	T	0.40 ± 0.06	0.36 ± 0.05	0.37 ± 0.04	**0.022**	**0.032**	0.702	**0.020**
	I	0.41 ± 0.05	0.39 ± 0.07	0.38 ± 0.03	0.345	0.672	0.132	0.265
	N	0.43 ± 0.05	0.41 ± 0.12	0.41 ± 0.03	0.413	0.405	0.956	0.355

SCP, superficial capillary plexus; DCP, deep capillary plexus; CVI, choroid vessel index; S, superior region; T, temporal region; I, inferior region; N, nasal region. P^*^, control versus Group 1; *P*^**+**^, control versus Group 2; *P*^#^, Group 1 versus Group 2; *p*, the differences among the three groups. Bold *p*-value represents < 0.05.

**Figure 4:**
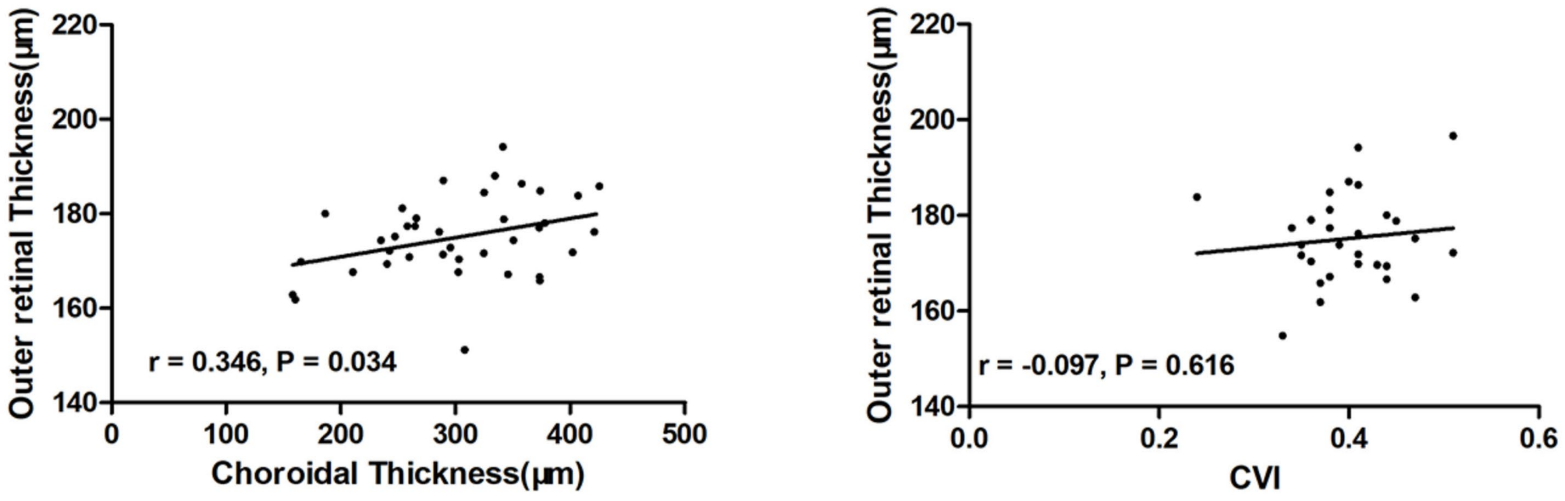
Analysis of correlation between outer retinal thickness and choroidal index in patients with unilateral nAMD. The outer retinal thickness was significantly correlated to choroidal thickness in patients with AMD.

There was no significant difference in the inner retinal thicknesses among the three groups (*p* > 0.05In Group 2, outer retinal thickness in the C, S, and T regions was significantly decreased compared to the control group (*p* = 0.048), (0.004), and (0.003), respectively) and Group 1 (*p* = 0.004), (0.005), and (0.014), respectively; Table 3). Meanwhile, compared to the controls, the total retinal thickness of Group 2 in the S (*p* = 0.034), T (*p* = 0.021), and I (*p* = 0.024) regions were lower (Table 3). Compared to the controls, the choroidal thicknesses of Group 1 tend to be decreased without significance, while the Group 2 were decreased in almost all regions (*p* = 0.001–0.022, Table 3) except for the T region. The outer retinal thickness was significantly correlated to choroidal thickness in patients with AMD (Figure 4).

**Table 3:** The thickness (μm) of retina and choroid among the three groups.

Layers	Regions	Control	Group 1	Group 2	P^*^	*P*^+^	*P*^#^	*p*
Inner retina	C	62.4 ± 13.7	68.9 ± 10.4	66.9 ± 10.0	0.375	0.928	0.396	0.627
	S	167.2 ± 11.4	165.3 ± 9.7	162.8 ± 15.0	0.497	0.094	0.419	0.242
	T	147.7 ± 10.4	147.1 ± 12.1	143.9 ± 13.4	0.914	0.072	0.160	0.176
	I	163.3 ± 8.8	166.7 ± 9.4	158.3 ± 14.4	0.831	0.077	0.196	0.194
	N	157.4 ± 8.9	160.5 ± 11.4	154.6 ± 15.0	0.815	0.463	0.420	0.680
Outer retina	C	186.4 ± 14.1	191.1 ± 10.3	178.3 ± 14.8	0.145	**0.048**	**0.004**	**0.015**
	S	170.4 ± 10.1	171.7 ± 8.1	159.5 ± 14.5	0.621	**0.004**	**0.005**	**0.006**
	T	173.4 ± 9.3	174.7 ± 8.0	162.6 ± 12.3	0.878	**0.003**	**0.014**	**0.012**
	I	166.3 ± 11.1	166.6 ± 5.2	158.8 ± 9.2	0.786	0.138	0.142	0.247
	N	175.6 ± 9.7	178.6 ± 7.3	170.8 ± 8.5	0.486	0.347	0.166	0.375
Total retina	C	249.1 ± 25.5	268.1 ± 22.5	245.0 ± 17.7	0.072	0.999	0.116	0.165
	S	336.8 ± 18.5	338.1 ± 16.6	322.4 ± 20.3	0.996	**0.034**	0.078	0.083
	T	321.1 ± 18.2	323.6 ± 12.9	307.8 ± 15.6	0.985	**0.021**	0.052	**0.049**
	I	330.1 ± 19.8	334.6 ± 10.4	317.1 ± 14.2	0.737	**0.024**	**0.031**	**0.044**
	N	332.9 ± 17.3	341.0 ± 15.4	326.3 ± 17.2	0.406	0.302	0.116	0.284
Choroid	C	347.4 ± 89.5	316.4 ± 66.9	286.8 ± 83.3	0.157	**0.011**	0.280	**0.031**
	S	348.1 ± 88.9	330.5 ± 65.4	294.1 ± 83.0	0.416	**0.022**	0.181	0.072
	T	345.0 ± 95.5	321.1 ± 64.2	298.7 ± 72.5	0.283	0.056	0.421	0.136
	I	340.5 ± 98.1	318.4 ± 78.5	272.5 ± 86.7	0.356	**0.001**	0.128	**0.034**
	N	328.8 ± 93.1	301.9 ± 73.9	266.1 ± 88.1	0.244	**0.013**	0.215	**0.040**

C, central region; S, superior region; T, temporal region; I, inferior region; N, nasal region. P^*^, control versus Group 1; *P*^+^, control versus Group 2; *P*^#^, Group 1 versus Group 2; *p*, the differences among the three groups. Bold *p*-value represents < 0.05.

## Discussion

This study used SS-OCTA to investigate macular microstructure and microvasculature in patients with dry AMD and those in earlier stages of the disease. We found that the total retina tended to be thinner in Group 2. In fact, the changes in the outer retina made the most significant contribution to the retinal alterations in patients with dry AMD. Therefore, there were also significant differences between the dry AMD group and the control group in the C region of outer retina. However, the inner retina counteracted this change, resulting in no significant difference in total retinal thickness in the C region. Compared to the controls, the densities of the SCP and DCP were significantly decreased in Group 2 in some regions. The choroidal thickness and CVI displayed a decreased gradient among the controls, Group 1, and Group 2. In addition, outer retinal thickness was significantly correlated to choroidal thickness in AMD patients.

Early AMD undoubtedly begins with imbalances within the photoreceptor/RPE/choriocapillaris/choroid system, as most previous reports have described (2, 24, 25). In addition, drusen and other sub-RPE deposits (basal laminar deposit and basal linear deposit, also referred to as diffuse drusen) are diagnostic and predictive of AMD (24). The thinning of the retina in early AMD was detected in a previous study along with apoptosis of RPE (26). The choroid was also significantly thinner in patients with dry AMD. The vascular density of the choriocapillaris tend to be decreased in early AMD, as demonstrated in a previous study of cell biology (27–29). Decreased subfoveal choroidal thickness and choriocapillaris and choroidal vessel density in the contralateral eyes of nAMD patients was detected in another study (30). As the main blood supply of the outer retina, the choroid seemed to be impaired earlier than the retina itself. This was consistent with previous research that the loss of endothelial cells of the choriocapillaris is one of the earliest detectable events in AMD (5). At the same time, a significant correlation between outer retinal thickness and choroidal thickness in AMD patients in our study was also a strong piece of evidence. Using the OCTA, we observed a statistically significant reduction in SCP and DCP in Group 2 compared with the control group.

In the current study, there are some limitations. Firstly, we did not describe the exact layer of the outer retina, which is more sensitive to macular degeneration. Secondly, we used simple choroidal thickness to detect the blood supply of outer retina. Further study should describe all four layers of the choroid. Lastly, a longitudinal study with a larger sample size is needed to confirm retinal and choroidal changes from subclinical to clinical AMD.

In conclusion, CVI and choroidal thickness were slightly decreased in patients without clinically diagnosed AMD; this reduction was more pronounced in eyes with dry AMD. Moreover, outer retinal thickness and microvascular density of the inner retina were decreased in patients with dry AMD. This indicates that an impaired choroid might promote changes in the outer retinal microstructure, resulting in a risk of progression to fully developed AMD, which will be demonstrated in a future longitudinal study. SS-OCTA might be useful in evaluating microstructural and blood supply disorders of the outer retinal layers in eyes at high risk of progression to AMD.

## Data Availability

The raw data supporting the conclusions of this article will be made available by the authors, without undue reservation.
